# No-Needle Jet Intradermal Aminolevulinic Acid Photodynamic Therapy for Recurrent Nodular Basal Cell Carcinoma of the Nose: A Case Report

**DOI:** 10.1155/2011/790509

**Published:** 2010-10-18

**Authors:** Daniel Barolet, Annie Boucher

**Affiliations:** ^1^RoseLab Skin Optics Laboratory, 3333 Graham Boulevard, Suite 206, Montreal, Quebec, Canada H3R 3L5; ^2^Dermatology Division, Department of Medicine, McGill University, Montreal, Quebec, Canada H3G 1Y6

## Abstract

Photodynamic therapy (PDT) with aminolevulinic acid (ALA) to treat nodular basal cell carcinoma (BCC) has been shown to be beneficial. The success rate of ALA-PDT in the treatment of nodular BCC is dependent on optimal penetration of the photosensitizing agent and subsequent PpIX production. To enhance topical delivery of drugs intradermally, a needleless jet injection (NLJI), which employs a high-speed jet to puncture the skin without the side effects of needles, was used in one patient with recurrent BCC of the nose. Photoactivation was then performed using red light emitting diode [CW @ *λ* 630 nm, irradiance 50 mW/cm^2^, total fluence 51 J/cm^2^] for 17 minutes. Excellent cosmesis was obtained. Aside from mild crusting present for six days, no other adverse signs were noted. Clinically, there was no recurrent lesion up two years postintervention. Additional studies in larger samples of subjects are needed to further evaluate this promising technique.

## 1. Introduction

Basal cell carcinoma (BCC) is the most frequent type of skin cancer in humans [1, 2]. Nodular variant is the most common form and usually presents as a round, pearly, flesh-colored papule with overlying small blood vessels. As it expands, it often ulcerates centrally, leaving a raised, pearly border with telangiectases. They are often seen in sunlight-exposed areas and on actinic damaged skin. Although it is rarely life-threatening, if left untreated, it can produce local destruction, cause bleeding, and be disfiguring. Moreover, large and longstanding tumours may metastasize into regional lymph nodes and surrounding tissues and bones. Hence, these lesions must be treated readily.

A wide range of treatment options exist to treat nodular BCCs (e.g., cryotherapy, Imiquimod 5% cream, laser, and radio therapies), yet, surgical excision and Mohs micrographic surgery remain the gold standard treatments for this condition. Unfortunately, postoperative scars are often visible and undesirable on areas such as the face. Photodynamic therapy (PDT) is a treatment modality that is increasingly used in dermato-oncology as an alternative to surgery to treat nodular BCC. PDT is based on photochemical reactions mediated through the interaction of light, oxygen, and a photosensitizing molecule (e.g., aminolevulinic acid [ALA]) induced protoporphyrin IX (PpIX) production, during which cytotoxic reactive oxygen species are formed causing damage (necrosis, apoptosis) to the target structures. Results from clinical research suggest that topical PDT is efficacious in nodular BCC [3–6] and conveys the advantage over surgery of better cosmesis [7–11].

The comparative outcome of excision surgery versus topical-PDT (ALA or methyl aminolevulinate [MAL]) was documented in three randomized clinical trials in primary nodular BCC [8–11]. In all studies, the lesions were prepared with superficial curettage or debridement. The photosensitizing agent (20% ALA cream or 160 mg/g of MAL cream) was then applied to the lesion area and predefined margin, with a light-occlusive dressing for an incubation period that lasted between 3 and 6 hours. Across the studies, different light sources were used to activate the photosensitizer in the red spectrum (570 to 730 nm), in order to improve tissue penetration, for a total dose of 75 J/cm^2^ or 125 J/cm^2^. Patients received either one or two cycles of PDT, and in some trials, PDT was repeated after 3 months if there was evidence of residual lesions. Short-term (3 months) results for clearance rate revealed no significant difference between topical-PDT and surgery. Long-term followups (up to five years), however, indicated superior lesional response of surgery over PDT overall, although PDT was also deemed efficacious and exhibited a more favourable cosmetic outcome. Differential efficacy of MAL-PDT versus ALA-PDT was not assessed in these trials but one further study conducted by Kuijpers et al. (2006) [12] did not observe any differences in short-term efficacy (8 weeks) between these agents, suggesting that both ALA and MAL can be equally used as topical photosensitizers in PDT for nodular BCC. Overall, the use of topical-PDT with red light appears to be a reasonable treatment option for nodular BCC, and in particular, in situations where surgery may be a suboptimal treatment choice [13, 14]. 

One possible limitation of PDT in nodular BCC is the penetration depth of the photosensitizer into the thick tumour volume, which can impact subsequent PpIX production levels and treatment efficacy [15, 16]. The application of ALA intralesionally, as opposed to topically, has been suggested as a mean to increase the penetration of photosensitizers. Recent research has reported higher fluorescence and PpIX levels after the intracutaneous administration of ALA in contrast to conventional topical application [17, 18]. The use of traditional needles, however, may lead to profound vascular compromise with possible vasoconstriction, deep purpura, necrosis, and infection and cause pain [19]. An alternative technique to enhance delivery of drugs intradermally is the needleless jet injection (NLJI) which employs a high-speed jet to puncture the skin and distribute the photosensitizer more evenly without the side effects of needles [20]. The potential of NLJI for the field of ALA-PDT has recently been highlighted by a preliminary *in vitro* investigation using a cross-linked hydrogel as a transparent skin model [21]. 

This case study aimed at assessing the *in vivo* human efficacy of NLJI ALA-PDT with red light (630 nm) in the treatment of a patient with recurrent nodular BCC of the nose.

## 2. Materials and Methods

### 2.1. Case Description

The patient was a 53-year old female type I Caucasian with recurrent nodular BCC of the nose after two unsuccessful excisional surgeries done by a qualified plastic surgeon with 5 mm margins. The diagnosis of nodular BCC was confirmed by histopathological examination. Her past medical history indicated varicose veins and face-lift surgery. She was not taking any medication prior to and after the procedure. The patient presented a fibrotic translucent lesion of 5,5 mm diameter with telangiectasia and central atrophy on the left ala of her nose. Given the recurrences after surgery, the size and location of the lesion, PDT with intradermal administration of ALA was considered for this patient. Data was collected in accordance with the Declaration of Helsinki and the Principles of Good Clinical Practice.

### 2.2. Procedure

**Figure 1:**
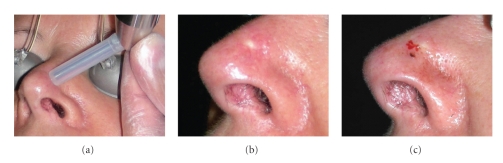
Experimental procedure. (a) Intradermal administration of ALA with a MadaJet medical injector; (b) lesion immediately post-Madajet and prior to the PDT procedure; (c) lesion after the PDT procedure.

An alcohol swab was used to clean the injection site before injection. A MadaJet Medical Injector (MADA Inc, Carlstadt, NJ) was used to deliver a high pressure spray of 0.4 cc, 20% ALA solution intradermally into the lesion (Figure 1). After an incubation period of one hour, the area was illuminated with red light emitting diode [CW @ *λ* 630 nm, irradiance 50 mW/cm^2^, total fluence 51 J/cm^2^] for 17 minutes. Before returning home, the patient was instructed on posttreatment skin care, which included applying topical fusidic acid cream twice a day, a plain moisturiser, sun avoidance, and the use of a sunscreen (SPF 30).

### 2.3. Clinical Assessments

Assessments of the lesion by means of visual analysis of digital photographs were carried out by two blinded physicians. Digital photographs (Canon Dual Flash EOS 10D, Canon, Tokyo, Japan with EX SIGMA 50 mm 1 : 2.8 macrolens, Sigma, Aizu, Japan) were taken at each visit maintaining as much as possible identical ambient lighting, pose, and camera angles. The lesion was assessed for clinical and morphological aspects. Raters assessed the lesion for *degree of improvement* from baseline to Month 6, 12, and 24 using a 5-point scale (0= none; 1= mild; 2= moderate; 3= good; 4= excellent). Raters also assessed *cosmetic outcome* at Month 6, 12, and 24 using a 4-point scale as follows: (1) excellent: no scarring, atrophy, or induration and slight or no redness or change in pigmentation compared with adjacent skin; (2) good: no scarring, atrophy, or induration and moderate redness or increase in pigmentation compared with adjacent skin; (3) fair: slight to moderate occurrence of scarring, atrophy, or induration; (4) poor: extensive occurrence of scarring, atrophy, or induration [22]. In addition, raters were asked to assess the *overall cosmetic outcome* using a 4-point scale (1= poor; 2= fair; 3= good; 4= excellent) at Month 6, 12, and 24.

### 2.4. Adverse Effects Monitoring

Adverse reactions were assessed at each visit, including signs of erythema, oedema, hematoma, ulcer scaling/crusting, bronzing, textural changes, hyperpigmentation, and hypopigmentation.

### 2.5. Patient Satisfaction

At the last followup visit, the patient was asked to rate her satisfaction level with the treatment results.

## 3. Results and Discussion

**Figure 2:**
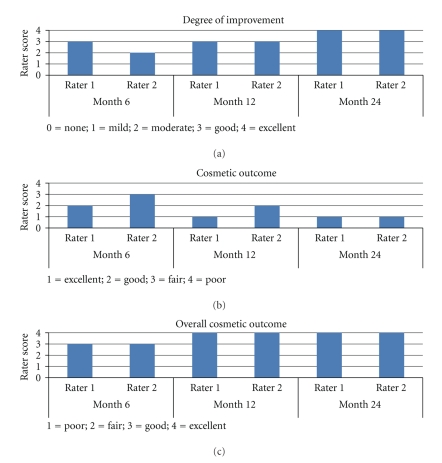
Results from the clinical assessments by two blinded physicians for (a) the *degree of improvement* from baseline, (b) the c*osmetic outcome*, and (c) *overall cosmetic outcome* at Month 6, 12, and 24.

**Figure 3:**
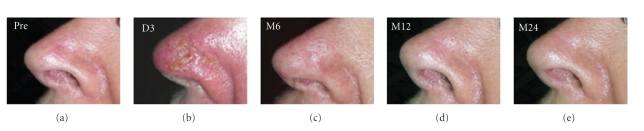
Treatment area pre-treatment (Pre), at day 3 (D3) and month 6 (M6), Month 12 (M12), and Month 24 (M24) post-PDT.

Lesion evaluations by two blinded clinicians revealed significant improvement for clinical and morphological aspects over time. The *degree of improvement* from baseline was rated as being *moderate* to *good* (scores of 2 and 3) at Month 6, *good* (scores of 3) at Month 12, and *excellent* (scores of 4) at Month 24 (Figure 2(a)). C*osmetic outcome *was deemed progressively better between Month 6 and Month 24, at which timepoint it was appraised as *excellent* (scores of 1), defined as no scarring, atrophy, or induration and slight or no redness or change in pigmentation compared with adjacent skin (Figure 2(b)). Moreover, the o*verall cosmetic outcome* was judged to be *good* (scores of 3) at Month 6 and *excellent* (scores of 4) at Month 12 and 24 (Figure 2(c)). Finally, clinical appraisal revealed that there was no recurrent lesion up two years postintervention. Figure 3 depicts digital photographs of the treatment area pretreatment and up to Month 24 post-PDT. 

The procedure was generally well tolerated by the patient, aside from a slight but bearable sensation of heat during photoactivation. With the exception of mild crusting present for six days, no other adverse signs, such hematoma or ulcer, were noted. Clinically, there was no recurrent lesion up to Month 24. Histological confirmation of BCC clearance was not obtained as the patient did not give consent to the skin biopsy procedure. The patient's satisfaction level with the treatment was extremely high.

## 4. Conclusion

Nodular BCC is a common form of skin cancer and must be treated readily as it can invade surrounding areas and cause significant destruction and disfigurement. In the present case, ALA-PDT with NLJI was used to directly deliver the photosensitizer into the lesion, to enhance PDT effects. This approach proved to be remarkably effective and well tolerated with no unusual adverse reactions and no recurrence two years postintervention in this patient with a large and high risk lesion. 

The selection of this technique had several advantages over more conventional methods for this particular patient. For one, this method did not necessitate mechanical impairment of the skin or removing the stratum corneum which can create discomfort for patients and complicate future outpatient treatments. In addition, unlike previously reported data with intracutaneous administration of ALA with traditional needles, no perceptible vascular compromise leading to necrosis was observed in the present case [19]. Moreover, the patient was able to avoid Mohs micrographic and extensive reconstructive surgery of the nose. 

Such a delivery method may be considered for recurrent lesions confined to surgically tricky or complex anatomical areas in selected patients. This technique may not be suitable, however, to treat large areas with multiple lesions at once, where standard PDT and other approaches or their combination would be more appropriate. The choice of the best possible procedure for a given patient is dependent on the type, size, and location of the lesion. It should be noted that, in general and for the nodular subtype in particular, surgical excision remains the treatment of choice to treat BCCs [13]. 

Whatever the chosen approach, biopsies are recommended to confirm the diagnosis and determine the histological subtype, as well as to document lesion clearance post-intervention. The post-PDT histological confirmation was unfortunately not possible for this patient. While BCCs are well recognised clinically, and results from clinical trials generally show agreement between clinical and histological assessments of tumours [23], the need for cautious long-term followups in this patient is warranted. 

The results obtained in this case are encouraging; however, supplementary studies in larger samples of patients and in the long-term are needed to further evaluate this promising technique. Future controlled studies are also necessary in order to determine if other photosensitizers, light sources, and wavelengths would procure added benefits to patients with an optimal clinical efficacy/side effects ratio. Other types of lesions, such as superficial BCC, Bowen's, and squamous cell carcinoma, could also potentially benefit from the use of this novel method and could be the object of additional trials.
